# O.5.2-1 Return to running after childbirth: barriers and facilitators

**DOI:** 10.1093/eurpub/ckad133.242

**Published:** 2023-09-11

**Authors:** Megan James, Diane Crone, Lynne Evans, Victoria Stiles, Isabel Moore

**Affiliations:** Cardiff Metropolitan University, UK; Cardiff Metropolitan University, UK; Cardiff Metropolitan University, UK; University of Exeter, UK; st20154529@outlook.cardiffmet.ac.uk; Cardiff Metropolitan University, UK

## Abstract

**Purpose:**

To investigate barriers to and facilitators of returning to running after childbirth, to inform the design of a return to running intervention.

**Methods:**

The study adopted a mixed-methods design, using an e-survey for data collection with 503 postpartum runners (age: 34.8 ± 3.8 years) within 24 months after childbirth. The e-survey comprised closed- (quantitative) and open-ended (qualitative) questions. The closed-ended questions related to demographics, birth details, physical activity and running behaviour. The open-ended questions focussed on respondents’ experiences and opinions on the barriers and facilitators that ‘helped’, ‘hindered’ or ‘would have helped’ them to return to running. Quantitative data were presented as medians with interquartile ranges (IQR) and proportions. Free text responses to the open-ended questions were analysed using thematic analysis.

**Results:**

On completion of the e-survey, participants were a median time of 10 months post-birth (IQR: 5-16). Of the cohort, 81% had returned to running (n = 405), with a median time of 12 weeks (IQR: 8-16) to first post-birth run. However, only just over a third of those who had returned to running had returned to at least their pre-pregnancy level (n = 146). Prominent themes for what ‘helped’, ‘hindered’ and ‘would have helped’ mothers return to running included psychological factors, physical factors that affected recovery, presence or absence of advice, degrees of childcare, time and access to running, as well as availability of training programmes and social support.

**Conclusions:**

The key barriers to and facilitators of returning to running after childbirth were psychological, physical, and social in nature, with a number of possible interactions between them. The findings suggest the need for the development of an interdisciplinary intervention to address these factors holistically to support postpartum women in returning to running. Future research, utilising more in-depth methods such as interviews or focus groups, should be undertaken to increase understanding of some of these barriers and facilitators further. In particular, exploration of the possible interactions between these factors would allow for the design of an intervention that is appropriate to these relationships.

**Support/Funding Source:**

Funded PhD studentiship from the Economic and Social Research Council.

